# Circulating Tumor Cells Enriched by the Depletion of Leukocytes with Bi-Antibodies in Non-Small Cell Lung Cancer: Potential Clinical Application

**DOI:** 10.1371/journal.pone.0137076

**Published:** 2015-08-28

**Authors:** Jian Yin, Yi Wang, Hanlu Yin, Wenping Chen, Guangfu Jin, Hongxia Ma, Juncheng Dai, Jiaping Chen, Yue Jiang, Hui Wang, Zhian Liu, Zhibin Hu, Hongbing Shen

**Affiliations:** 1 Department of Epidemiology & Biostatistics, School of Public Health, Nanjing Medical University, Nanjing, China; 2 Department of Respiratory Diseases, Nanjing Chest Hospital, Nanjing, China; 3 Jiangsu Key Laboratory of Cancer Biomarkers, Prevention and Treatment, Cancer Center, School of Public Health, Nanjing Medical University, Nanjing, China; Graduate Institute of Toxicology, TAIWAN

## Abstract

**Background:**

It has been considered that the detection methods for circulating tumor cells (CTCs) based on epithelial cell adhesion molecule (EpCAM) underestimate the number of CTCs and may miss a metastatic subpopulation with cancer stem cell (CSC) properties. Therefore, we investigated EpCAM-positive and -negative CTCs in non-small cell lung cancer (NSCLC) patients at different stages, assessed the clinical value of these CTCs and explored their capacity in the following CSC model.

**Methods:**

CTCs were enriched by the depletion of leukocytes with bi-antibodies using a magnetic bead separation technique and then identified by the expression of EpCAM and cytokeratin 7 and 8 using multi-parameter flow cytometry. We determined the distribution of CTCs classified by the expression of EpCAM in 46 NSCLC patients with stages I to IV, assessed the diagnostic value of these CTCs by longitudinal monitoring in 4 index patients during adjuvant therapy and characterized the stemness of these CTCs by the expression of CXCR4 and CD133 in 10 patients.

**Results:**

EpCAM-negative (E-) CTCs were detected to be significantly higher than EpCAM-positive (E+) CTCs in stage IV (*p* = 0.003). The patients with the percentage of E-CTCs more than 95% (*r* > 95%) were detected to be significantly increased from 13.3% in stage I-II to 61.1% in stage IV (*p* = 0.006). Kaplan–Meier analysis indicated that the patients with *r* > 95% had significantly shorter survival time than those with *r* ≤ 0.95 (*p* = 0.041). Longitudinal monitoring of CTCs indicated that the patients with a high percentage of E-CTCs in the blood were not responsive to either chemotherapy or targeted therapy. Further characterization of CTCs revealed that a stem-like subpopulation of CXCR4+CD133+ CTCs were detected to be significantly more prevalent in E-CTCs than that in E+CTCs (*p* = 0.005).

**Conclusions:**

The enrichment of CTCs by the depletion of leukocytes with bi-antibodies is a valuable method for estimating the number of CTCs, which can be potentially applied in predicting the prognosis, monitoring the therapeutic effect of NSCLC patients and further analyzing the biology of CTCs.

## Introduction

Lung cancer is the leading cause of cancer-related death [1], and approximately 85% of lung cancer cases are non-small cell lung cancer (NSCLC). Although systematic treatment has been improved, the overall 5-year survival rate is only 10–20% [2]. The primary reason for the low survival rate is distant metastasis of tumor cells. In the metastatic cascade, circulating tumor cells (CTCs) have been considered to be key participants in the formation of distant metastases [3]. A previous study showed that CTCs expressing epithelial cell adhesion molecule (EpCAM) are detectable in stage IV NSCLC patients and are a novel prognostic factor for this disease [4]. However, it has been suggested that the methods based on the expression of EpCAM underestimate the number of CTCs and may miss a metastatic subpopulation of CTCs with cancer stem cell (CSC) properties [5, 6]. A recent study reported that CTCs are detected 2 times more effectively by ISET (isolation by size of epithelial tumor cells) than those by CellSearch, and that a subpopulation of CTCs, which did not express EpCAM (i.e., E-CTCs), can be detected in the blood of NSCLC patients [7]. Another study has also shown that the enumeration of these cells is much higher than that of CTCs captured by CellSearch [8]. Until now, the clinical value and biology of these E-CTCs has been unclear, and a recent publication has indicated that future studies should include the detection of E-CTCs [9].

CTCs undergoing epithelial-mesenchymal transition (EMT) have been considered to play an important role in the formation of neoplasms [5]. EMT can generate cells with stem cell properties [10]. During EMT, the expression of EpCAM in tumor cells will be down-regulated. A previous study reported that the SDF-1/CXCR4 axis plays an important role in mediating cell migration and survival after a TGF-β-induced EMT [11]. However, it remains unclear whether these (or any) CTCs with down-regulated EpCAM have an increased metastatic seeding potential or heightened resistance to systemic therapy, and, as recently indicated, a greater prognostic value [12]. Our previous study revealed that CXCR4-expressing CTCs were detected in the blood of solid tumor patients [13]. A recent study reported that the up-regulation of CXCR4 is functionally crucial for the maintenance of stemness in drug-resistant NSCLC cells [14]. Therefore, it is necessary to characterize the stemness of E-CTCs.

CTCs are rare in the blood of tumor patients, and studies on the biology of CTCs are limited by the low concentration and yield of CTCs [9]. Although previous studies have shown that the ISET technique is more effective in capturing E-CTCs in NSCLC [7, 8], a recent study reported that lung tumors grow faster and shed a significant number of lethal metastatic cells at small sizes [15]. Therefore, an “unbiased” isolation method of CTCs is desirable. We have previously established a negative enrichment of CTCs [16], which is based on the depletion of leukocytes using a magnetic bead separation technique and subsequent detection by multi-parameter flow cytometry. In the present study, we improved this technique by depleting leukocytes with bi-antibodies to improve the purity of CTCs for their characterization and future application in molecular analysis. By this method, we determined the distribution of CTCs classified by the expression of EpCAM in 46 NSCLC patients with stages from I to IV, investigated their diagnostic value in systematic therapy by longitudinal monitoring of these CTCs in 4 index patients, assessed the prognostic value of these CTCs and characterized the stemness of these CTCs by measuring the expression of CXCR4 and CD133.

## Materials and Methods

### Ethics Statement

Written informed consent was obtained from all patients and healthy volunteers before participating in the study. Blood sample collection and analyses were approved by the institutional review board of Nanjing Medical University. The design and performance of the current study involving human subjects were clearly described in a research protocol.

### Study population and preparation of blood samples

Peripheral blood samples from 46 NSCLC patients were collected from January to November in 2013 in Nanjing Chest Hospital and Jiangsu Cancer hospital. The patients’ characteristics are listed in Table 1. Blood was drawn after discarding the first 2 mL to avoid potential skin cell contamination from venipuncture, and then processed within 4 hours after collection. The blood samples from 4 index patients were also collected at the end of each period of chemotherapy or targeted therapy immediately, and the therapeutic effects of patients were assessed according to Response Evaluation Criteria in Solid Tumors.

**Table 1:** Characteristics of NSCLC patients.

Characteristics	Number of patients	%
**Total number of cases**	46	
**Age at diagnosis, years**		
**Median**	58	
**Range**	41–88	
**Sex**		
**Female**	19	41.3
**Male**	27	58.7
**Histologic subtype**		
**Adenocarcinoma**	39	84.8
**Squamous cell carcinoma**	7	15.2
**Tumor Stage at diagnosis**		
**I**	10	21.7
**II**	6	13.1
**III**	8	17.4
**IV**	22	47.8

### CTC enrichment

The cell solutions from blood samples were prepared as previously described [16]. CTCs were then enriched by the depletion of leukocytes with bi-antibodies according to the following steps: first, based on Leukocyte common antigen CD45, human Whole Blood CD45 Depletion Kit (EasySep, Stem Cells Technologies, Inc., Vancouver, BC, Canada) was used as the first step for leukocyte depletion; second, based on leukocyte surface antigen CD53, cells were stained by PE mouse anti-human CD53 antibody (BD Bioscience, USA), washed with PBS and then further depleted with PE positive selection Kit (EasySep, Stem Cells Technologies, Inc., Vancouver, BC, Canada) as the second step for the leukocytes. The procedure was conducted following the manufacturers’ instructions with minor modifications.

### Flow cytometry

After enrichment, CTCs were divided equally into 2 tubes and stained with human anti-EpCAM, anti-CK7/8, anti-CD45 (BD Bioscience, USA) in the first tube and corresponding isotype control antibodies in the second tube. To explore the biology of CTCs in stemness, the enriched cells were together stained with human anti-CXCR4 (BD Bioscience, USA) and anti-CD133 (Miltenyi Biotec GmbH, Germany) (in the first tube) and the corresponding isotype control antibodies (in the second tube) in 10 samples. The final concentration of each antibody was 10μg/mL. A FACS AriaIII system (BD Biosciences) was performed to determine the expression of all markers, and data were analyzed using FlowJo 7.2.5 software (Tree Star, Ashland, OR, USA). Cells with the expression of the above markers were gated by the corresponding isotype control based on distinct division between them. CTCs were defined as Cytokeratin+CD45-CD53- cells.

### Spiking experiment

The human lung cancer cell line PC9 (high expression of EpCAM and Cytokeratin) was contributed by the Cancer Center of Nanjing Medical University and cultured in high glucose DMEM medium containing 10% FBS, 2 mM L-glutamine, 100 U/mL penicillin and 100 μg/mL streptomycin at 37°C in a humidified 5% CO_2_ atmosphere. PC9 cells were harvested according to the same procedure as previously described [16]. Zero, 10, 50, 100 and 500 PC9 cells were spiked into 2 mL of blood from healthy volunteers. PC9 cells were then enriched according to the method described above. After enrichment, CTCs were divided equally into 2 tubes and stained with human anti-EpCAM, anti-CK7/8, anti-CD45 and the corresponding isotype control antibodies. In flow cytometric analysis, the percentage of CTCs in all remained cells (including leukocytes and CTCs) after enrichment was defined as the purity of CTCs. Recovery and purity were calculated to evaluate the performance of the method, which were determined as follows: Recovery = n_1_/n_2_×100%, Purity = n_1_/(n_1_+n_3_)×100% (n_1_ was the number of PC9 cells found after enrichment; n_2_ was the number of PC9 cells spiked into 2 mL of blood; n_3_ was the number of leukocytes found after enrichment). The spiking experiment in each condition was repeated three times.

To check our CTC platform is still workable, PC9 cell line with low expression of EpCAM was obtained according to the following steps: cells were seeded at a density of 5 × 10^5^ cells in four 6cm-dishes and incubated in 5% CO_2_, 95% air at 37°C till its density reached 80%. Cultured cells were maintained in serum-free DMEM (0.1% BSA, 2 mM L-glutamine, 100 U/mL penicillin and 100 μg/mL streptomycin) at 37°C in a humidified 5% CO_2_ atmosphere for overnight, and then stimulated by 5ng/mL rhTGF-β1 (PeproTech, USA) in the same medium for 3 days. The spiking experiments were proceeded using this type of PC9 cells (named PC9-EMT) according to the same procedure described as above.

### Cutoff value

The baseline of E+ and E-CTCs were determined by their enumeration in the blood samples from 11 healthy volunteers. The cutoff values of E+ and E-CTCs were calculated by the upper limit of the mean value of CTCs plus Standard Deviation (SD). If the two types of CTCs were both under the cutoff values, this patient would be defined as having undetected CTCs and would be excluded from statistical analysis. If only one type of CTC was under the cutoff value, this type of CTC would be treated as 0 for convenience in statistical analysis. The percentage of E-CTCs was calculated according to the total number of E+ and E-CTCs. If the percentage of E-CTCs was higher than 95% (*r* > 95%), this patient would be considered to have absolute majority of E-CTCs in the blood.

### Statistical analysis

Data analysis was carried out with Stata statistical packages (Stata Corporation, College station, TX, USA). Mann-Whitney and χ^2^ tests were used to compare the two groups. Cox proportional hazards model was performed using SPSS (Statistical Package for the Social Sciences) v. 16.0 (SPSS, Inc, Chicago, IL) to assess the correlation between the survival time of patients and the percentage of CTCs. *P* values < 0.05 were considered to present significant differences.

## Results

### Evaluation of the CTC enrichment method

**Fig 1:**
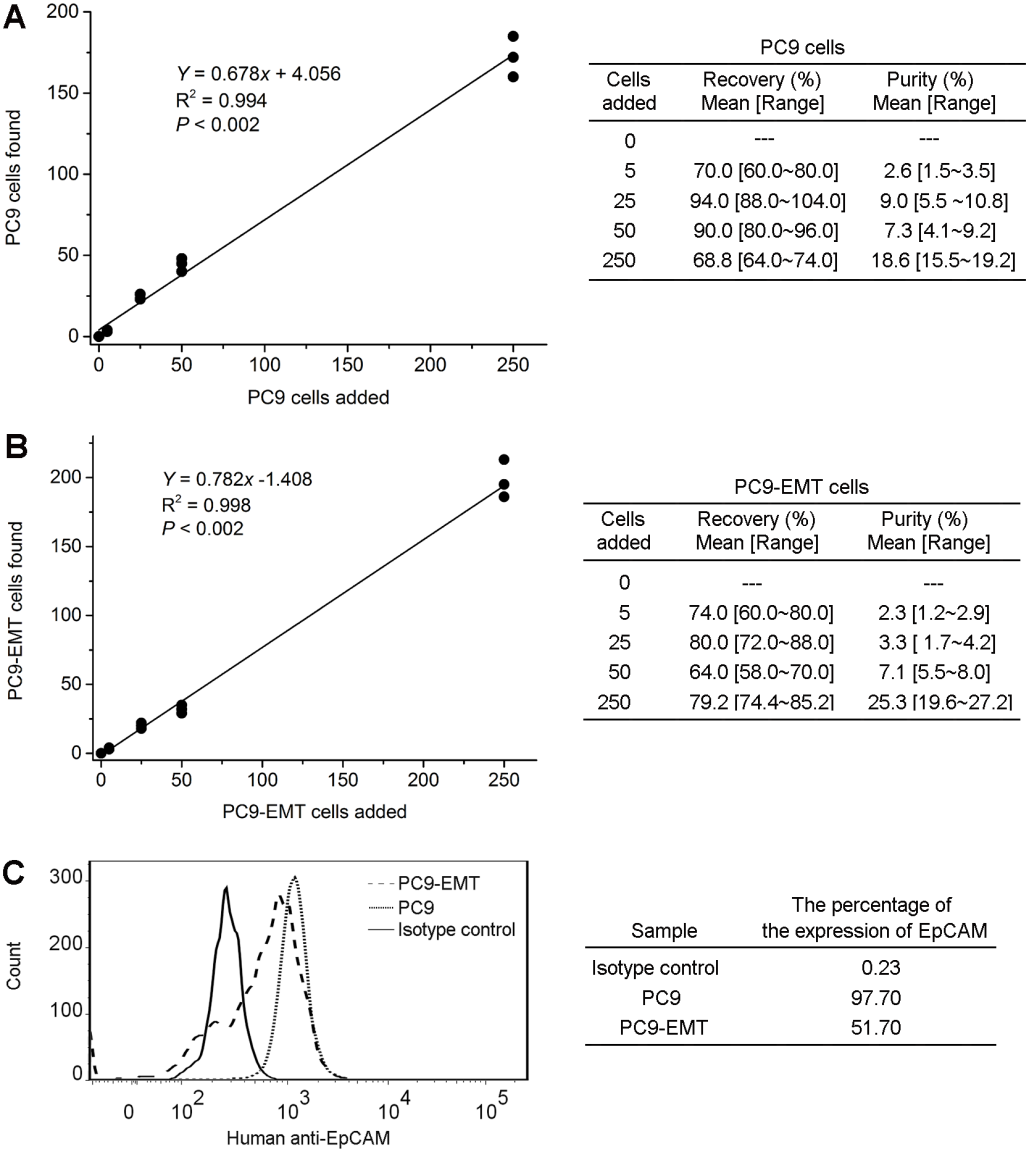
**C**alibration curves in spiking experiments using (**A**) PC9 cell line and (**B**) PC9-EMT cells; (**C**) FACS analysis for the expression of EpCAM in PC9 and PC9-EMT cells stimulated by 5ng/mL TGF-β for 3 days.

After stimulation by TGF-β for 3 days, the expression of EpCAM in PC9 cells decreased from 97.7% to 51.7%. Using our method in spiking experiments, the mean value of the recovery / purity of PC9 and PC9-EMT cells were 80.7% / 9.5% and 74.3% / 9.5%, respectively. The calibration curve are shown in Fig 1. There is a linear relationship with the slope at 0.678 (0.782) between the logarithms of PC9 cells (PC9-EMT cells) found and PC9 cells (PC9-EMT cells) added.

The cutoff values of E+ and E-CTCs were determined in 11 blood samples from healthy volunteers. E+CTCs were detected in only one sample, and E-CTCs were detected in 8 samples. The mean value of CTCs + SD was 0.09 + 0.30 and 1.18 + 0.98/mL of blood for E+ and E-CTCs, respectively. Therefore, zero and 2 were defined as the cutoff values for E+ and E-CTCs, respectively.

In 46 blood samples, CTCs were undetected in five samples (1 in stage II and 4 in stage IV) due to both E+ and E-CTCs under the corresponding cutoff values. In 41 blood samples with detected CTCs, The mean value [range] of the purity of CTCs was 10.6% [0.1%-51.1%]. Of these samples, 36.6% (15 of 41) had a purity of CTCs over 10.0%. The maximum purity of CTCs was 51.1%. Representative results of the FACS analysis are provided in S1 Fig.

### Distribution of CTCs in patients

**Fig 2:**
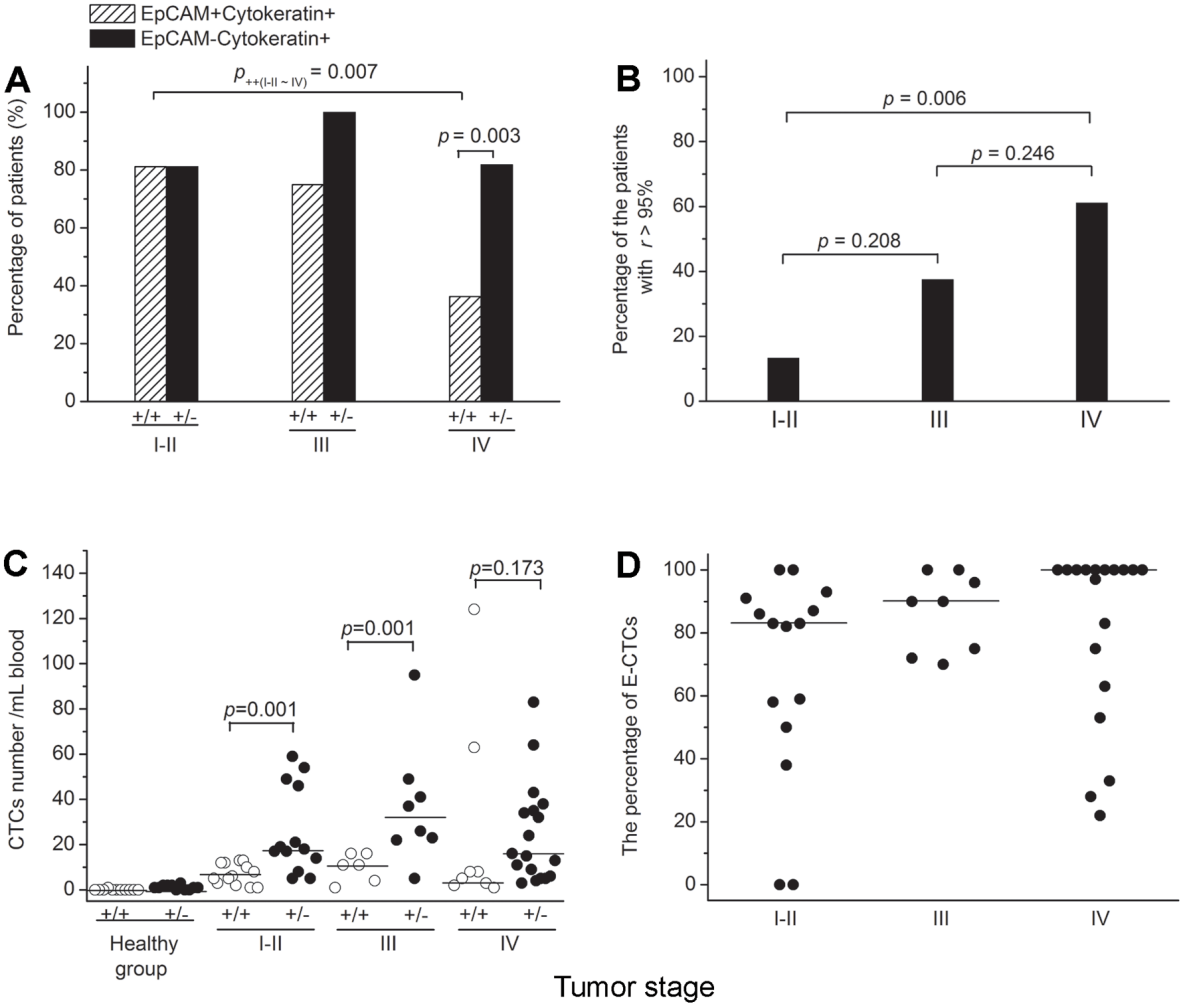
Distribution of CTCs classified by the expression of EpCAM in NSCLC patients. (**A**) Distribution of the detection rates of E+ and E-CTCs in patients at different stages; (**B**) Percentage of detected patients with a percentage of E-CTCs higher than 95% (*r* > 95%); (**C**) Distribution of the number of E+ and E-CTCs in patients at different stages; (**D**) Distribution of the Percentage of E-CTCs in different stages. (+/+, Cytokeratin+EpCAM+; +/-, Cytokeratin+EpCAM-).

The detection rates of E+ and E-CTCs are shown in Fig 2A. E+CTCs were detected in 13 of 16 (81.3%) stage I-II, 6 of 8 (75.0%) stage III and 8 of 22 (36.4%) stage IV patients. E-CTCs were detected in 13 of 16 (81.3%) stage I-II, 8 of 8 (100%) stage III and 18 of 22 (81.8%) stage IV patients. E+CTCs were determined to be significantly lower in stage IV patients than in stage I-II patients (*p* = 0.007). E+CTCs and E-CTCs were detected to be significantly different in stage IV patients (*p* = 0.003). Furthermore, 2 of 15 (13.3%) stage I-II, 3 of 8 (37.5%) stage III and 11 of 18 (61.1%) stage IV patients were detected to have an absolute majority of E-CTCs in their blood (*r* > 95%). The frequency of patients with *r* > 95% was detected to be significantly higher in stage IV than in stage I-II patients (*p* = 0.006, Fig 2B).

According to the cutoff values, CTCs were detected in 41 patients and the distribution of the number of E+ and E-CTCs is shown in Fig 2C. The median values [Interquartile Range, IR]/mL blood of E+ and E-CTCs were 6.0 [2.5–12.0] and 18. [11.0–47.5] in stage I-II patients, 11.0 [3.25–16.0] and 31.5 [22.5–31.5] in stage III and 6.5 [2.25–49.25] and 15.5 [5.75–35.75] in stage IV patients, respectively. There was a significant difference in the number of E+ and E-CTCs in both stage I-II and III (*p* = 0.001 for both), but not in stage IV because the number of E+CTCs was increased in several patients. The distribution of the percentage of E-CTCs is shown in Fig 2D. The median values [IR] of the percentage of E-CTCs were 83.0% [50.0%-91.0%] in stage I-II, 90.0% [72.8%-99.0%] in stage III and 100.0% [60.5%-100.0%] in stage IV patients. Although a high percentage of E-CTCs was found in all stages, there was not a significant difference.

### Longitudinal monitoring of CTCs in adjuvant therapy

**Fig 3:**
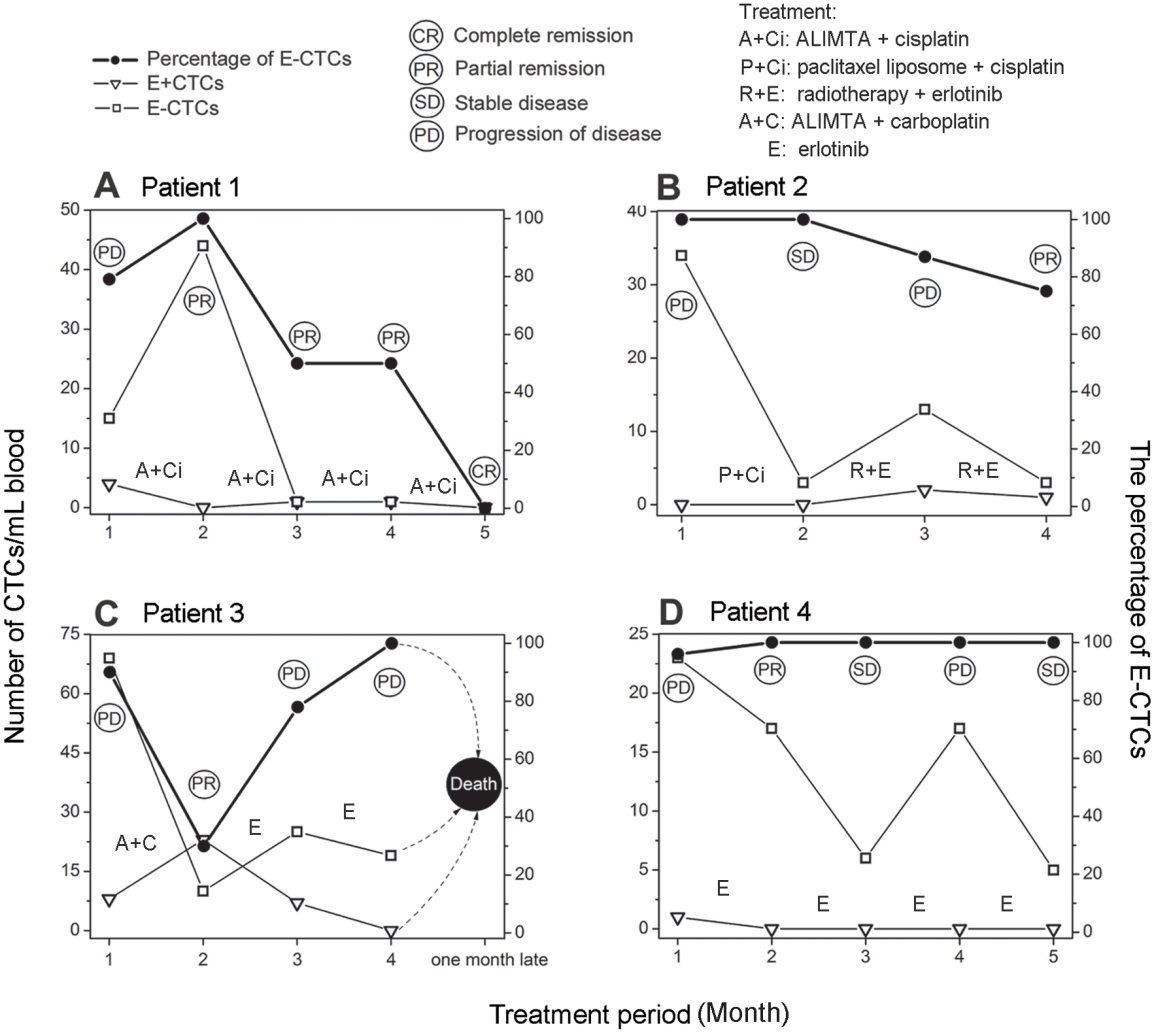
Longitudinal monitoring of CTCs in adjuvant therapy. (**A**) Gefitinib and then ALIMTA + cisplatin treatment; (**B**) Paclitaxel liposome + cisplatin and then radiotherapy + erlotinib treatment; (**C**) ALIMTA + carboplatin and then erlotinib treatment; (**D**) Erlotinib treatment. Each period of treatment was one month.

E+ and E-CTCs were dynamically monitored in 4 index patients, and the results are shown in Fig 3. Patient 1 (Fig 3A and S2 Fig), who had progressive disease after initial treatment with Gefitinib, showed the disease from partial to complete remission in the treatment with ALIMTA (pemetrexeddisodium) + cisplatin. The number of E+CTCs decreased from 4 to 0/mL of blood, but the number of E-CTCs increased from 15 to 44/mL of blood in the first period and decreased to near 0/mL blood in the last three periods. The percentage of E-CTCs was 79% at the beginning, slightly increased to 100% during the first period, decreased to 50% and maintained at this level in the following two periods, but was undetected in the last period.

Patient 2 (Fig 3B and S3 Fig), who had progressive disease after initial treatment with paclitaxel liposome + cisplatin, showed stable disease during the first period but had progressive disease during the second period due to a brain metastasis. After treatment with radiotherapy + erlotinib, this patient showed partial remission of disease. The number of E-CTCs decreased from 34 to 3/mL of blood in the first period, increased to 13 /mL of blood in the second period and then decreased to 3/mL of blood in the last period, whereas the number of E+CTCs showed insensitive change from 0, 0, 2 to 1/mL of blood during the therapy. The percentage of E-CTCs was maintained at the high level (100%) during the first period, and then decreased to 87% and 75% in the last two periods.

Patient 3 (Fig 3C and S4 Fig), who had progressive disease after initial treatment with ALIMTA + carboplatin, showed partial remission during the first period but had progressive disease during the second period of the treatment with erlotinib. This patient died one month later. The number of E-CTCs decreased from 69 to 10/mL of blood in the first period, and increased to 25 and 19/mL of blood in the last two periods. The number of E+CTCs showed a disordered change from 8 to 23/mL of blood in the first period, and then decreased to 7/mL of blood and undetectable in the last two periods. The percentage of E-CTCs was maintained at a high level (90%) in the beginning, decreased to 30% along with a response to partial remission of disease and then increased to 78% and 100% following the progression of disease.

Patient 4 (Fig 3D and S5 Fig), who had progressive disease in the initial diagnosis, showed partial remission, stable disease, progressive disease and stable disease from the first to fourth period in the treatment with erlotinib. The number of E-CTCs showed a fluctuant change from 23, 17, 6, 17 to 5 /mL of blood, whereas E+CTCs were nearly undetected at all times. The percentage of E-CTCs was maintained near to 100% at all times.

### Follow-up

**Fig 4:**
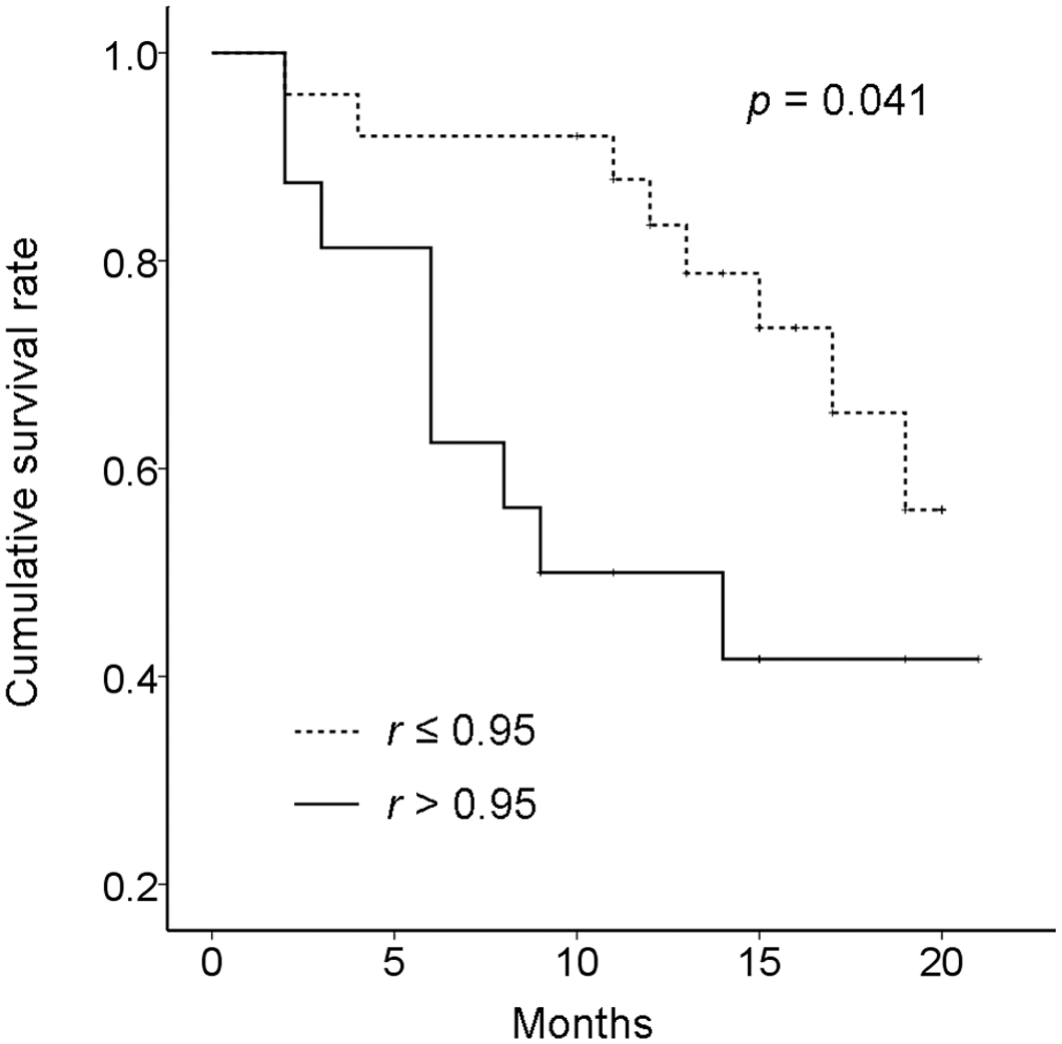
Kaplan-Meier survival analyses of E+ and E-CTCs in NSCLC patients.

All patients had follow-up records from 10 to 20 months depending on the sampling time. Seventeen patients died due to a distant metastasis (stages I-II: 3 cases; stage III: 3 cases; stage IV: 11 cases). In the 17 cases, 4 patients were excluded from the statistical analysis because CTCs were undetected. In 41 patients with detectable CTCs, 9 of 16 patients with absolute majority of E-CTCs in their blood (*r* > 95%) and 8 of 25 patients with *r* ≤ 95% died. Kaplan—Meier analysis indicated that the patients with *r* > 95% had significantly shorter survival time than those with *r* ≤ 95% (*p* = 0.041, Fig 4).

### Characterization of CTCs in stemness

**Fig 5:**
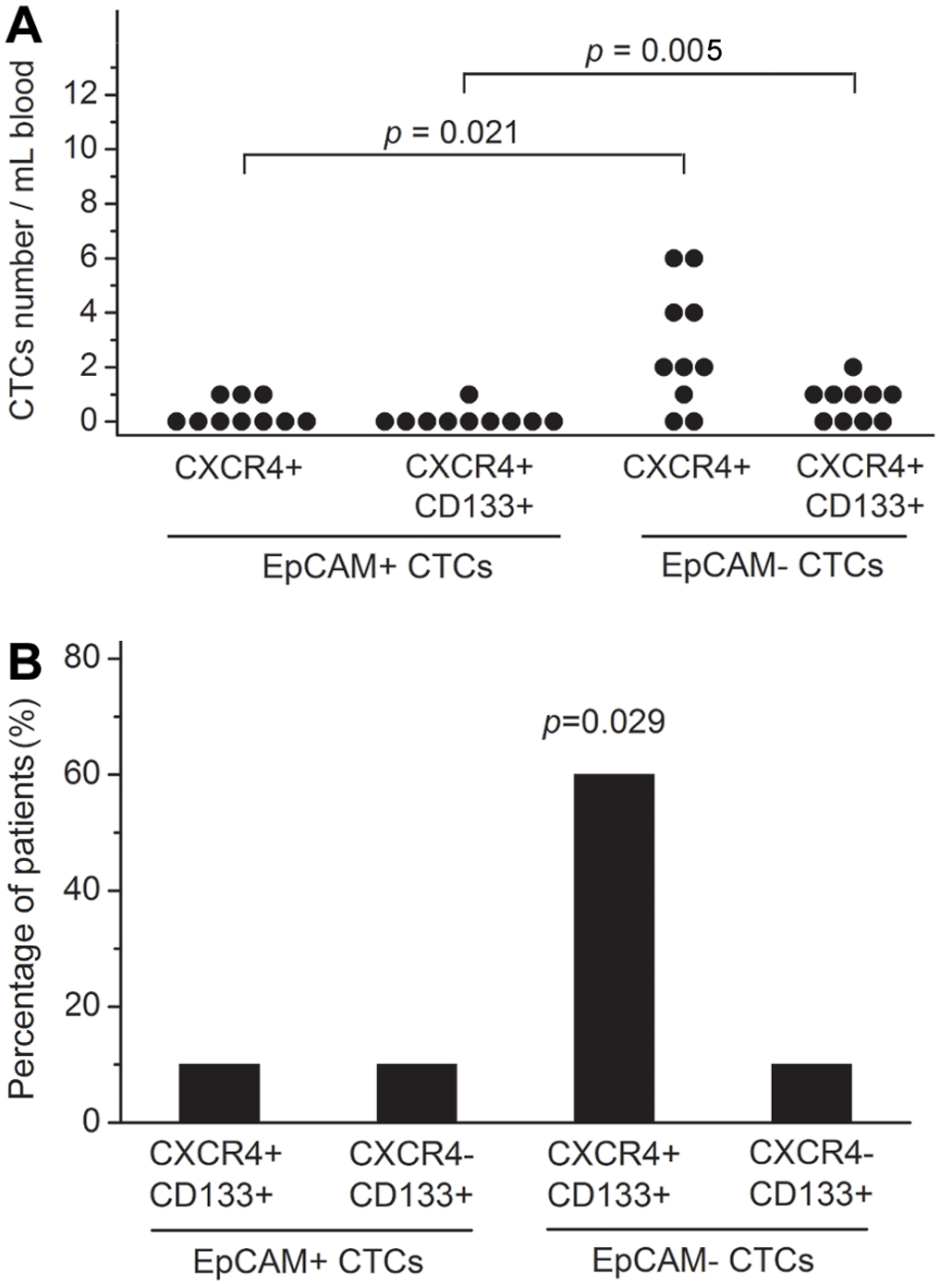
Characterization of CTCs in stemness. Distribution of (**A**) the number of CXCR4+ and CXCR4+CD133+ cells and (**B**) the positivity detection rates of CXCR4+CD133+ cells in CTCs classified by the expression of EpCAM in 10 NSCLC patients.

To explore the capacity of CTCs following CSC model, we determined the expression of CXCR4 and CD133 on E+ and E-CTC, respectively, in 10 patients. CXCR4 was detected in 8/10 of patients within the subgroup of E-CTCs, whereas it was detected in only 3/10 of the patients within the subgroup of E+CTCs. Both CXCR4 and CD133 were detected in 6/10 of patients within the subgroup of E-CTCs, whereas they were detected in only 1/10 of patients within the E+CTCs subgroup. CXCR4+CTCs and CXCR4+CD133+CTCs were detected to be significantly higher in E-CTCs than in E+CTCs (*p* = 0.021 and 0.005, respectively, Fig 5A). Additionally, the expression of CD133 was found to be significantly higher in CXCR+E-CTCs than in other types of CTCs (*p* = 0.029, Fig 5B). Interestingly, in the same individuals, the number of CTCs with the expression of CXCR4 and/or CD133 was always more within the subgroup of E-CTCs than that of E+CTCs. No patient could be found that the expression of CXCR4 and/or CD133 were detected only in E+CTCs but not in E-CTCs.

## Discussion

CTCs can be used as a prognostic marker [17, 18]. However, the small number and low concentration of CTCs has limited study of the biology of CTCs [9, 19]. Due to the heterogeneity of CTCs, it is difficult to capture all types of CTCs by EpCAM-based methods. A more reasonable strategy is to deplete leukocytes. Using our improved method, the purity of CTCs was increased by 10 times in spiking experiments, and up to 50 times higher in blood samples compared with a previously reported method based on magnetic nanobeads [16]. Using this method, the mean value of the purity of CTCs could be up to 10% in spiking experiments and 10.6% in the blood samples of patients, which also supported its further application in molecular analysis. This method is comparable for the detection of CTCs with and without the expression of EpCAM. In the results, 36.4% (for E+CTCs) and 81.8% (for E-CTCs) of patients had stage IV disease. These data are consistent with the published results that E+CTCs were detected in only 23~32% of advanced NSCLC patients by CellSearch and 80% of those by size isolation strategy [4, 7]. This method is specific for the identification of CTCs with and without expression of EpCAM. Both E+ and E-CTCs were detected to be much lower in the blood samples from healthy volunteers relative to those in patients, which was also consistent with a previously published study [20]. Although E+ and E-CTCs were undetected in five patients, it is possible that the enumeration of CTCs was lower than the cutoff values or absent from additional specific markers to identify the heterogeneous tumor cells in NSCLC [21, 22].

In the present study, we investigated the distribution of CTCs with and without the expression of EpCAM in NSCLC patients by an improved method. In the results, E+ and E-CTCs were detected to be significantly different only in stage IV patients, although they were not in stage I-II and III patients. Furthermore, the percentage of patients with an absolute majority of E-CTCs in the blood significantly increased from stage I-II to IV. Moreover, the results by Kaplan—Meier analysis indicated that the patients with an absolute majority of E-CTCs in the blood had a shorter survival time than those patients with a low percentage of E-CTCs. These results suggested that the percentage of E-CTCs could be used to predict the prognosis of NSCLC patients.

Presently, the diagnostic value of CTCs with and without the expression of EpCAM in NSCLC is unclear. In this study, we conducted longitudinal monitoring in 4 index patients during adjuvant therapy. The percentage of E-CTCs was found to be more accurate than the number of E+CTCs in the assessment of therapeutic effect on both chemotherapy and targeted therapy. Moreover, a low number of E-CTCs were associated with a positive response in patients. These results suggested that the percentage of E-CTCs may have potential as a marker in the monitoring and predicting of the therapeutic effect in NSCLC. It is possible that noncancerous cells may be included in E-CTCs [9], and we also did not find any clinical value in the simple enumeration of E-CTCs. This phenomenon was also consistent with the recently published results on chemotherapy in breast cancer [23]. However, our data indicated that a high percentage of E-CTCs may imply a strengthened ability of tumor cells in invasion and proliferation.

To explore the above biology of CTCs, we characterized the stemness of CTCs with different expression profiles of EpCAM following the results of our previous work [13]. Previous studies have suggested that a combination of CXCR4 and CD133 can be used as CSC marker [24, 25]. In our results, CXCR4+CD133+ CTCs were detected to be significantly more prevalent in E-CTCs than those in E+CTCs, and the number of E-CTCs with the expression of CXCR4 and/or CD133 was always more than that of E+CTCs in in the same individuals, which suggested that E-CTCs appeared to be more stem-like. This result may explain, at least in part, the poor outcome in the patients with overwhelming E-CTCs in the blood to some extent. Moreover, CXCR4 was up-regulated during TGF-β induced EMT. The potential relationship between the more stem-like E-CTCs and EMT should be further investigated. Although a relatively small number of patients were used in this study, these results contribute to a better understanding of the relationship between CSC and EMT in CTCs. Additional research with a large sample size and EMT identification are needed to clarify the status of this type of CTCs and its function in the tumor progression of NSCLC.

## Conclusion

Based on a technique of CTC enrichment by the depletion of leukocytes with bi-antibodies, this study assessed the clinical value of CTCs with and without EpCAM expression in NSCLC. Our data suggest that the percentage of E-CTCs significantly increased from stage I-II to IV and could be used to evaluate the distant metastasis, therapeutic effect and prognosis of patients in NSCLC. Furthermore, E-CTCs appeared to be more stem-like than E+CTCs. These results provide the first evidence for the potential clinical application of E-CTCs in NSCLC.

## Supporting Information

S1 Fig Representative FACS analysis on CTCs in the blood samples of 4 NSCLC patients. (TIF)

S2 Fig Computed Tomography (CT) images for patient 1 treated with Gefitinib and then ALIMTA (pemetrexeddisodium) + cisplatin. (TIF)

S3 Fig CT images for patient 2 treated with paclitaxel liposome + cisplatin and then radiotherapy + erlotinib. (TIF)

S4 Fig CT images for patient 3 treated with ALIMTA + carboplatin and then erlotinib. (TIF)

S5 Fig CT images for patient 4 treated with erlotinib. (TIF)
