# Supplementary figures and images for: Circulating Tumor Cells Enriched by the Depletion of Leukocytes with Bi-Antibodies in Non-Small Cell Lung Cancer: Potential Clinical Application
Source: PLoS One. 2015 Aug 28;10(8):e0137076. doi: 10.1371/journal.pone.0137076 (PMC4552861; doi:10.1371/journal.pone.0137076)

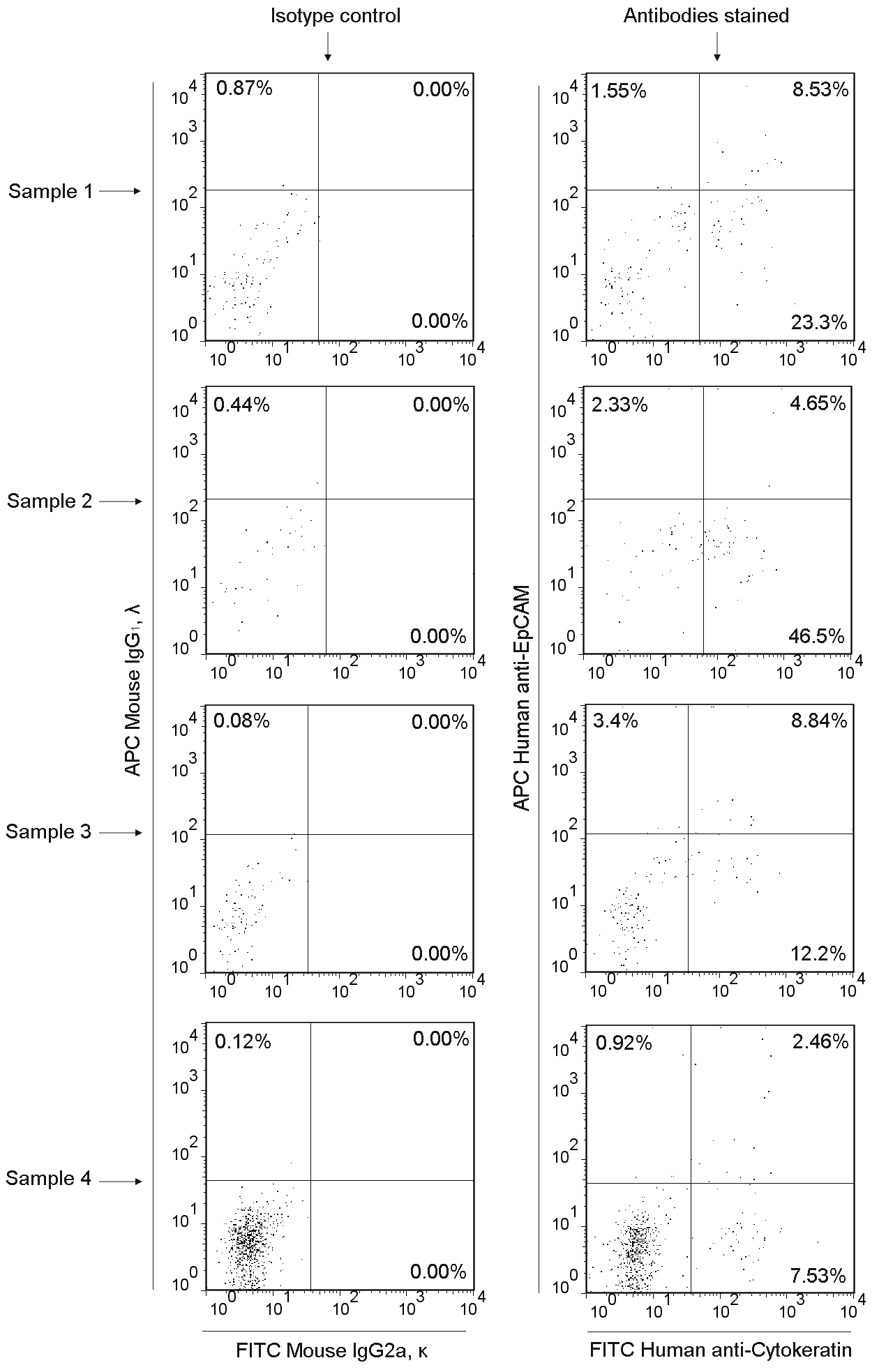

Supplement: S1 Fig — (TIF) [file pone.0137076.s001.tif]

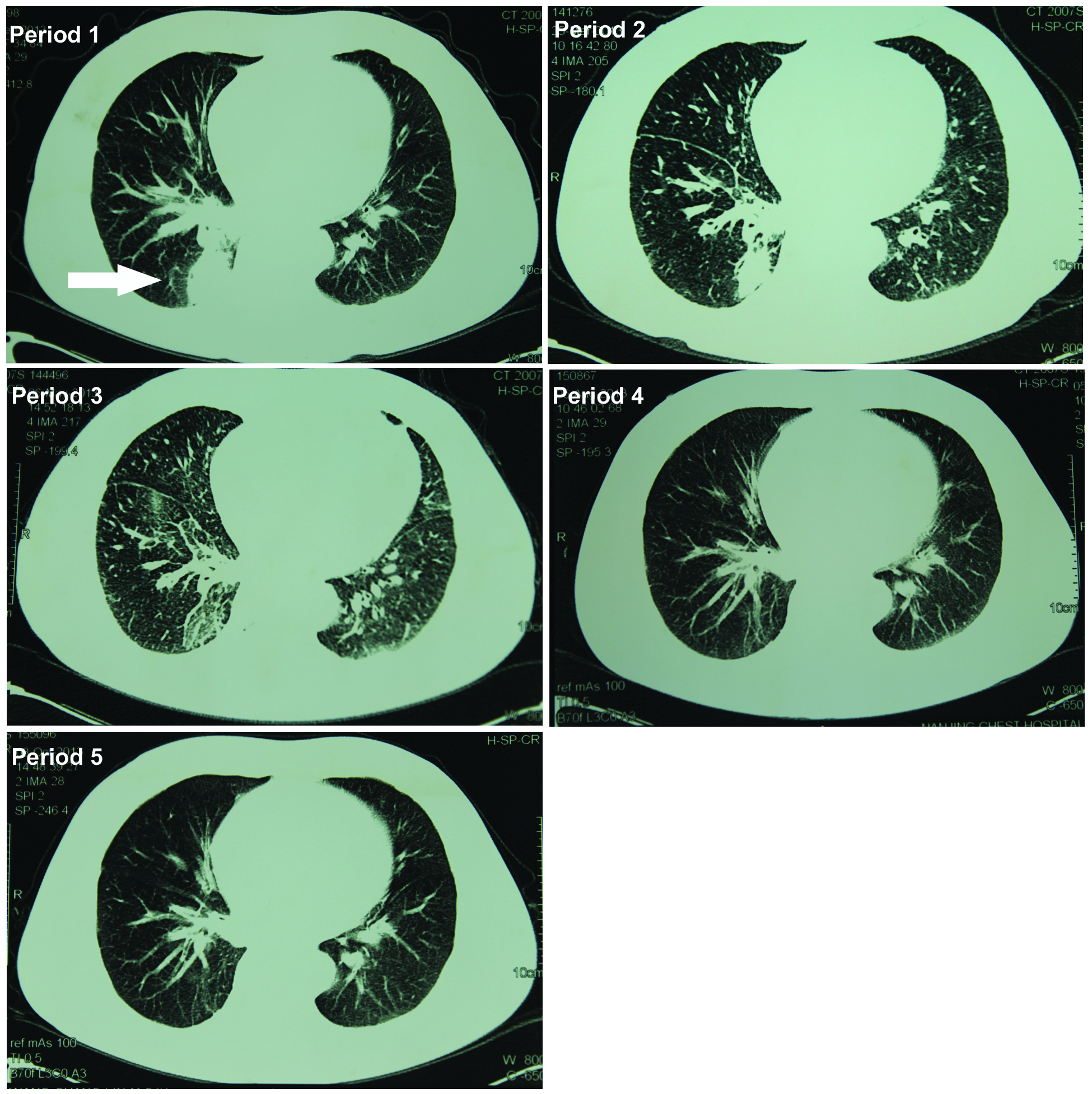

Supplement: S2 Fig — (TIF) [file pone.0137076.s002.tif]

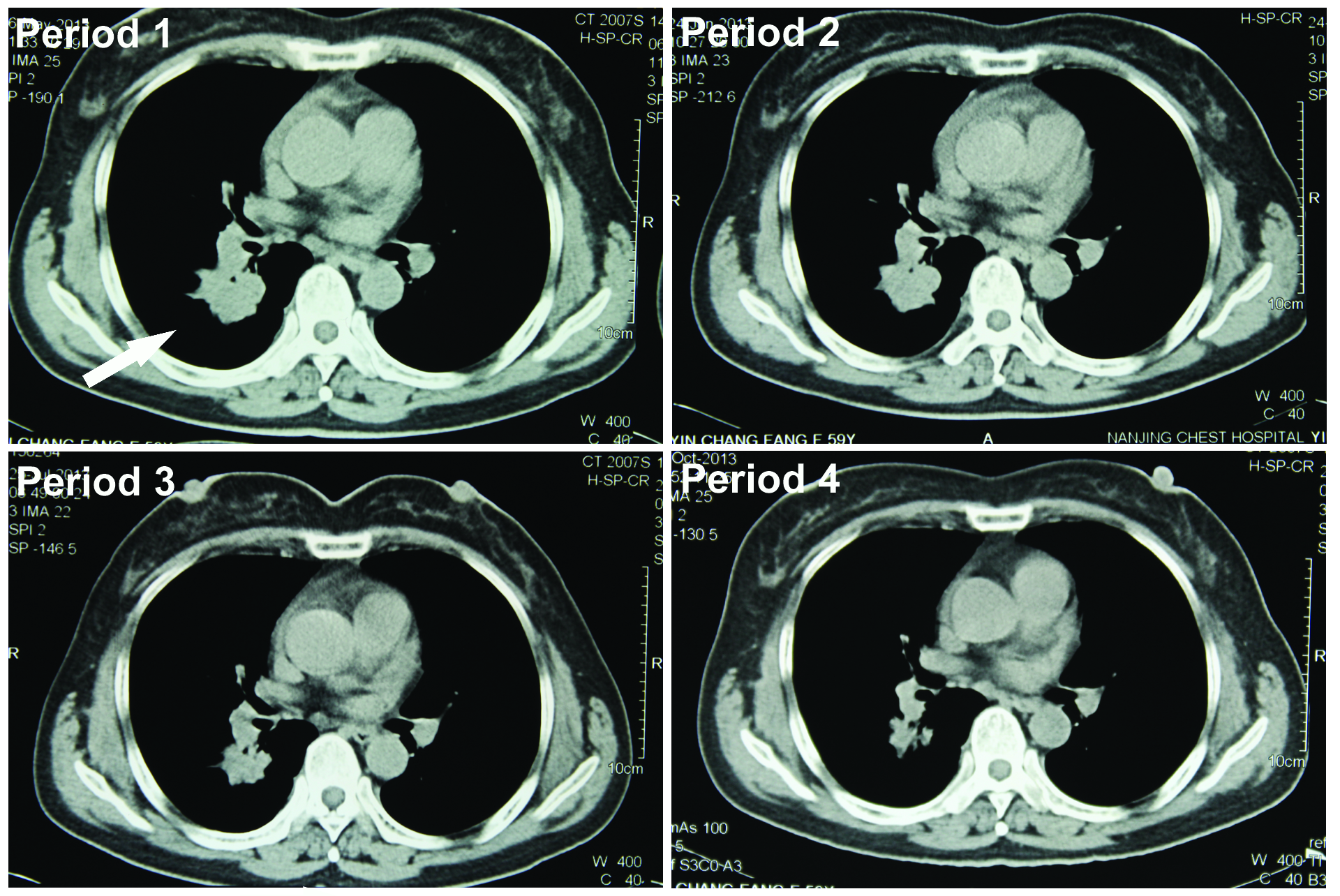

Supplement: S3 Fig — (TIF) [file pone.0137076.s003.tif]

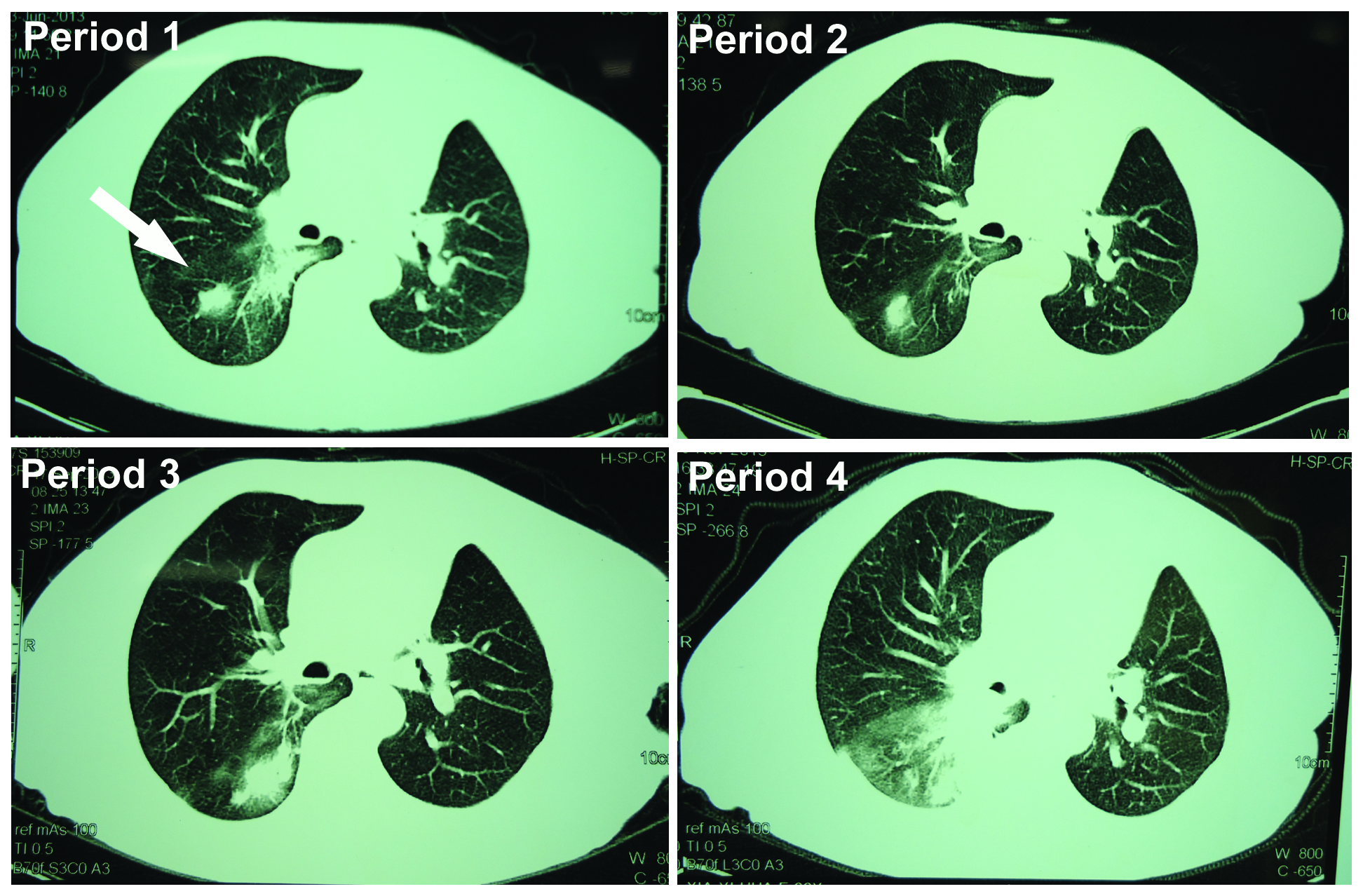

Supplement: S4 Fig — (TIF) [file pone.0137076.s004.tif]

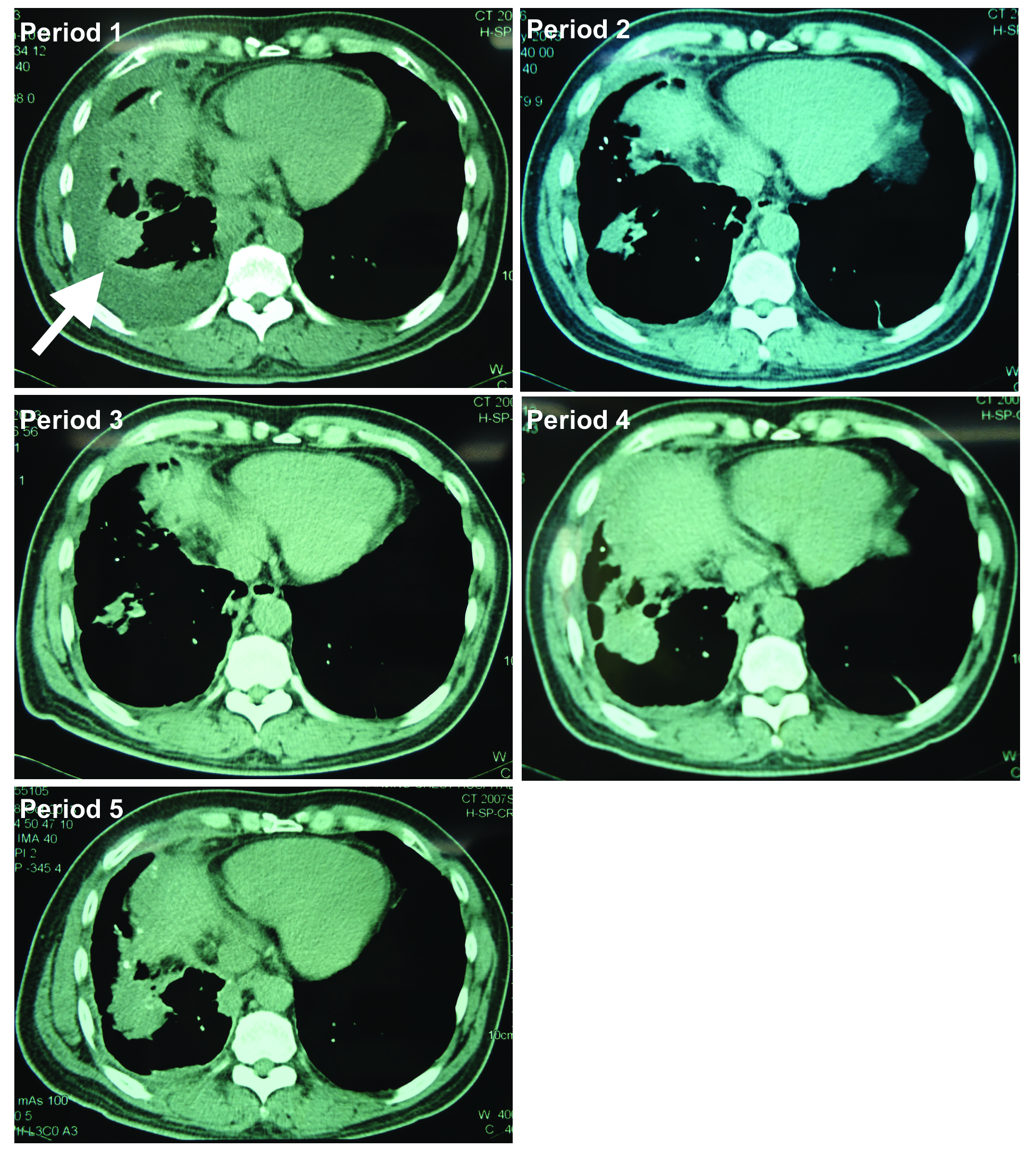

Supplement: S5 Fig — (TIF) [file pone.0137076.s005.tif]
